# Development and Preliminary Evaluation of a Multivariate Index Assay for Ovarian Cancer

**DOI:** 10.1371/journal.pone.0004599

**Published:** 2009-02-25

**Authors:** Suraj D. Amonkar, Greg P. Bertenshaw, Tzong-Hao Chen, Katharine J. Bergstrom, Jinghua Zhao, Partha Seshaiah, Ping Yip, Brian C. Mansfield

**Affiliations:** Correlogic Systems, Inc., Rockville, Maryland, United States of America; University Medical Center Rotterdam, Netherlands

## Abstract

**Background:**

Most women with a clinical presentation consistent with ovarian cancer have benign conditions. Therefore methods to distinguish women with ovarian cancer from those with benign conditions would be beneficial. We describe the development and preliminary evaluation of a serum-based multivariate assay for ovarian cancer. This hypothesis-driven study examined whether an informative pattern could be detected in stage I disease that persists through later stages.

**Methodology/Principal Findings:**

Sera, collected under uniform protocols from multiple institutions, representing 176 cases and 187 controls from women presenting for surgery were examined using high-throughput, multiplexed immunoassays. All stages and common subtypes of epithelial ovarian cancer, and the most common benign ovarian conditions were represented. A panel of 104 antigens, 44 autoimmune and 56 infectious disease markers were assayed and informative combinations identified. Using a training set of 91 stage I data sets, representing 61 individual samples, and an equivalent number of controls, an 11-analyte profile, composed of CA-125, CA 19-9, EGF-R, C-reactive protein, myoglobin, apolipoprotein A1, apolipoprotein CIII, MIP-1α, IL-6, IL-18 and tenascin C was identified and appears informative for all stages and common subtypes of ovarian cancer. Using a testing set of 245 samples, approximately twice the size of the model building set, the classifier had 91.3% sensitivity and 88.5% specificity. While these preliminary results are promising, further refinement and extensive validation of the classifier in a clinical trial is necessary to determine if the test has clinical value.

**Conclusions/Significance:**

We describe a blood-based assay using 11 analytes that can distinguish women with ovarian cancer from those with benign conditions. Preliminary evaluation of the classifier suggests it has the potential to offer approximately 90% sensitivity and 90% specificity. While promising, the performance needs to be assessed in a blinded clinical validation study.

## Introduction

Ovarian cancer is the deadliest gynecological cancer in the United States [1]. In 2008, an estimated 21,650 new cases of ovarian cancer will be detected. Early diagnosis is associated with a 92% 5-year survival rate, yet only 19% of ovarian cancers are detected early [1], [2]. The majority of cases detected are advanced stage disease where 5-year survival rates for women with regional malignancy and distant disease are 71% and 30% respectively. As a result, more than 15,000 women die from ovarian cancer in the US each year [1].

The early symptoms of ovarian cancer, which include pelvic and abdominal pain, urinary urgency and frequency, abdominal bloating, and difficulty eating are non-specific, and typical of many non-cancerous and benign conditions [3]. Therefore, diagnosis does not typically occur until the development of either a significant amount of abdominal fluid, or a pelvic mass, detected by physical examination or with radiologic evaluation [4]. A recent report has suggested that a unique combination of symptoms, if fully documented for each patient, may be more informative than previously recognized, although the findings remain to be validated [5]. Many reports indicate that the most commonly used imaging techniques – transvaginal sonography (TVS), positron-emission tomography (PET), magnetic resonance imaging (MRI), radioimmunoscintigraphy and computed tomography (CT) lack sufficient specificity to distinguish between benign and malignant ovarian disease [6]. Some recent studies have suggested that ultrasound alone, or in combination with other prognostic variables may be significantly more informative in the hands of a specialized ovarian ultrasound expert [7], [8], however, many patients do not have access to the skills of such specialists. Moreover, clear diagnosis usually necessitates, at minimum, surgical intervention in the form of laparotomy or laparoscopy. Therefore, an accurate, informative, yet non-invasive, test would be of clinical value.

There are no FDA-approved biomarkers for the diagnosis of ovarian cancer, or for the triage of women suspected of having ovarian cancer. Despite its widespread use, cancer antigen 125 (CA-125) is only FDA-approved for monitoring recurrence and therapeutic response [9]–[11]. In studies of women with known or suspected ovarian cancer, the reported sensitivities of CA-125 in detecting stage I and II cancers range widely from 29–75% and 67–100%, respectively. However, CA-125 is elevated in a wide variety of normal, benign and malignant conditions [12]–[14] and 86% of women presenting with abnormal CA-125 tests resolve in 3–6 months [15]. Many approaches have been taken to improve the predictive value of CA-125 through serial measurements [16], [17] or in combination with additional markers [18]–[21]. However, a simple and clinically practical ovarian cancer-screening tool remains elusive.

A recent study [22] described a panel of six markers – CA-125, prolactin, leptin, macrophage inhibitory factor (MIF), osteopontin and insulin-like growth factor II (IGF-II) that when combined had very high sensitivity (95.3%) and specificity (99.4%). The test is intended as a screen on high-risk women, however, the final performance characteristics were not assessed on high-risk women and included samples also used to build models which may have resulted in over-estimation of the classifier's performance. Moreover, inclusion and exclusion criteria for participants were not clearly defined, and the cancer and control samples were collected under different clinical settings, which can lead to bias in the sample set. Prolactin and IGF-II were each reported to be individually more informative than CA-125, in this study, but this is inconsistent with reports on other independent sample sets [23], [24]. In another study, Moore and colleagues utilized logistic regression to find marker combinations capable of differentiating between benign and malignant conditions in women with pelvic masses [25]. By combining HE-4 and CA-125, 76.4% sensitivity and 95% specificity was achieved. While promising, only 67 of the 233 samples were from individuals with ovarian cancer and only 15 of those from women with stage I and II cancers. In addition, reported performance was based on cross-validation results which lacked an independent holdout set of samples.

Ovarian cancer is a collection of diverse entities with more than 30 subtypes of malignancies, each with a distinctive histology, pathology and clinical behavior [26]. The diversity and low incidence of ovarian cancer hampers the search for biomarkers. In a separate, post-hoc analysis, of a subset of the samples used in the present study, we were unable to identify a single marker capable by itself of accurately predicting the presence of ovarian cancer [24]. In this present study, we describe the development and preliminary evaluation of a multi-analyte profile that can classify women suspected of having ovarian cancer, into those with and without ovarian cancer.

## Methods

### Sample Cohort

All but 20 samples were from the tissue-banking repository of the National Cancer Institute-funded Gynecologic Oncology Group (GOG; Columbus, OH; Table 1; Table S2). Written consent was obtained by the GOG for all participants and the GOG Institutional Review Board (IRB) approved the use of the samples in our study. These samples were collected from multiple sites, under protocols approved by the GOG IRB. Eligible patients were women scheduled for surgery with suspicion of having a gynecological cancer or scheduled for prophylactic surgery because of increased ovarian cancer risk (1st or 2nd degree relative with the disease). All samples, including those categorized as normals, post-surgery, were collected prior to any diagnostic or therapeutic intervention. Serum aliquots forwarded to Correlogic Systems, Inc.® (Rockville, MD) had been de-identified and encoded with a unique GOG identifier. Each sample was accompanied by a complete clinicopathology report, patient age and race, and a de-identified code denoting the collection site. Pathology was reviewed and confirmed by GOG pathologists to ensure consistency. Samples were selected from the GOG collection to balance patient age distribution, date of serum collection, and representation of cases and controls across collection sites. The remaining sera consisted of 20 samples from individuals with benign conditions from a Correlogic prospective collection, which uses a similar serum collection protocol. Written consent was obtained from all participants. Correlogic's “prospective” samples are being collected under IRB approval to support the development of a clinical test for ovarian cancer. The study population is women presenting with symptoms of ovarian cancer and scheduled for surgery. As such, disease status is confirmed by pathology following surgery. The 20 samples were withdrawn from the prospective collection in a manner to avoid introducing any bias into the remaining collection and as such were not deliberately selected to represent any particular population. The study was approved by the Western IRB (Olympia, WA) and by the IRB of each participating site.

**Table 1:** Demographics of study subjects.§ ‡

	Stage# I	Stage II	Stage III	Stage IV	Stage X	All OvCa	Normal	Benign†	Other Cancer*	All Non OvCa
**Number of Subjects (%)**	61 (34.7)	31 (17.6)	67 (38.1)	12 (6.8)	5 (2.8)	176 (100)	32 (17.1)	140 (74.9)	15 (8.0)	187 (100)
**Median Age (range)**	53.0 (29–80)	56.0 (39–85)	57.0 (42–87)	66.5 (28–78)	71.0 (52–80)	56.0 (28–87)	45.5 (29–72)	52.0 (15–88)	54.0 (27–89)	50 (15–89)
**Mean Age (SD)**	55.8 (11.0)	57.6 (10.3)	59.5 (11.8)	62.8 (14.7)	68.5 (11.8)	58.3 (11.6)	47.9 (9.5)	54.3 (14.4)	55.8 (16.7)	53.4 (14.1)
**Serous**	12	18	31	10	3	74 (42.0)	-	-	-	-
**Mucinous**	6	3	6	1	0	16 (9.1)	-	-	-	-
**Clear cell**	17	0	9	0	1	27 (15.3)	-	-	-	-
**Endometrioid**	22	8	13	1	1	45 (25.6)	-	-	-	-
**Mixed**	4	2	8	0	0	14 (8.0)	-	-	-	-

§ Two low malignant potential samples not tabulated.

‡ All samples were sourced from a single GOG study with the exception of 20 benign samples which were sourced from a Correlogic prospective collection as described in “Methods – Sample Cohort”. Only GOG samples are shown in this table.

* Other cancers consisted of 4 endometrial cancers, 7 cervical cancers, 3 colon cancers and 1 uterine cancer.

† The most common benign sample types were cystadenoma, endometrioma/endometriosis, cyst, Brenner tumor and adenofibroma.

# Staging based on FIGO, International Federation of Gynecology and Obstetrics. Stage X, staging not available. OvCa, ovarian cancer.

### Serum Processing, Storage, Handling and Shipment

Blood samples (5–20 ml) were collected into red top glass Vacutainer tubes (Becton-Dickinson, NJ), clotted for 30–180 minutes at 4°C, and then centrifuged at 3,500 g for 10 minutes at 4°C. Serum was decanted into cryotubes, and stored promptly at −80°C. Aliquots from storage were shipped to Correlogic on dry ice and stored immediately at −80°C. Frozen samples were warmed gently by hand until almost thawed, completed on ice, vortexed, aliquoted in 150 ul volumes and refrozen at −80°C. Finally, samples were shipped on dry ice to Rules-Based Medicine, Inc. (RBM; Austin, TX). An accompanying document provided a coded sample identification number and a specific order of analysis. The RBM analytical site was completely blinded to all sample details including disease status.

### Multiplex Immunoassays

The multiplexed immunoassays are described elsewhere [24]. Briefly, two rounds of multiplexed immunoassays were conducted at RBM in their Luminex-based CLIA-certified laboratory. Analytes were quantified by reference to 8-point calibration curves and machine performance was verified using three quality control (QC) samples for each analyte. QC samples were distributed relatively evenly across the dynamic range of the assay at low, medium and high levels and generally had coefficients of variance below 15%. Calibration standards and QC samples were in a complex plasma-based matrix to match the sample background and were analyzed in duplicate. In round one, a total of 204 analytes representing 104 antigens, 44 autoimmune and 56 infectious disease molecules were measured in 147 epithelial ovarian cancer samples (40 stage I, 23 stage II, 67 stage III, 12 stage IV, five unstaged) and 149 control samples (104 benign conditions, 29 normal healthy, 14 other cancers and two low malignant potential) using proprietary multiplexed immunoassays (Table S1). A second round of analysis was performed 86 days after the first analytical round, on the 104 antigens, using a second serum aliquot that had been subjected to an identical freeze/thaw history as the samples used in round one. Due to sample volume restrictions, 27 samples were not reanalyzed in round two. Thus, in round two, 132 ovarian cancer samples (30 stage I, 21 stage II, 65 stage III, 11 stage IV and five unstaged) and 135 controls (94 benign conditions, 28 normal healthy, 13 other cancers) were reanalyzed. In addition, a further 69 samples, not included in round one, were analyzed (21 stage I, eight stage II, 36 benign, three normal healthy and one colon cancer). For both rounds of analysis, the order of analysis was established to avoid any sequential bias due to disease presence or absence, subtype or stage of disease, patient age, or age of the serum sample. Generally, samples alternated between cases and controls.

### Data Handling

Since sera were analyzed at a previously optimized dilution, any sample exceeding the maximum concentration of the calibration curve was arbitrarily assigned the concentration of the highest standard, whereas those assayed below the minimum concentration of the calibration curve were assigned the value 0.0. A single assay (IL-1α) that showed no variation in expression across all samples was considered invariant/uninformative and removed from the extracted data set. The remaining data were then scaled by the biweight scale; a robust and efficient scaling mechanism that accounts for the variance within each of the individual assays [27]. A single scale for each assay was determined in a population-weighted manner. Any assay yielding a scale factor of zero was removed from the data set. The resulting data were then exported into individual files where each file represented the results of all qualified assays for a single sample.

### Modeling – “Out-of-Bag” Error Estimation and Bootstrap Validation

To minimize sample set bias and to aid in the assessment of intermediate models, we employed one-third “out-of-bag” (OOB) error estimation and an external 100-fold bootstrap validation with 10% holdout bootstraps. These bootstrap estimates allowed us to assess the potential value of many models using only the training data. In this way we were able to maintain the independence of the hold-out testing set of samples. Only after a specific classifier had been locked into a traceable document management system (DMS) were the hold-out testing set of data used to test performance of the selected model.

### Modeling – Proof-of-Principle Classifier

**Figure 1:**
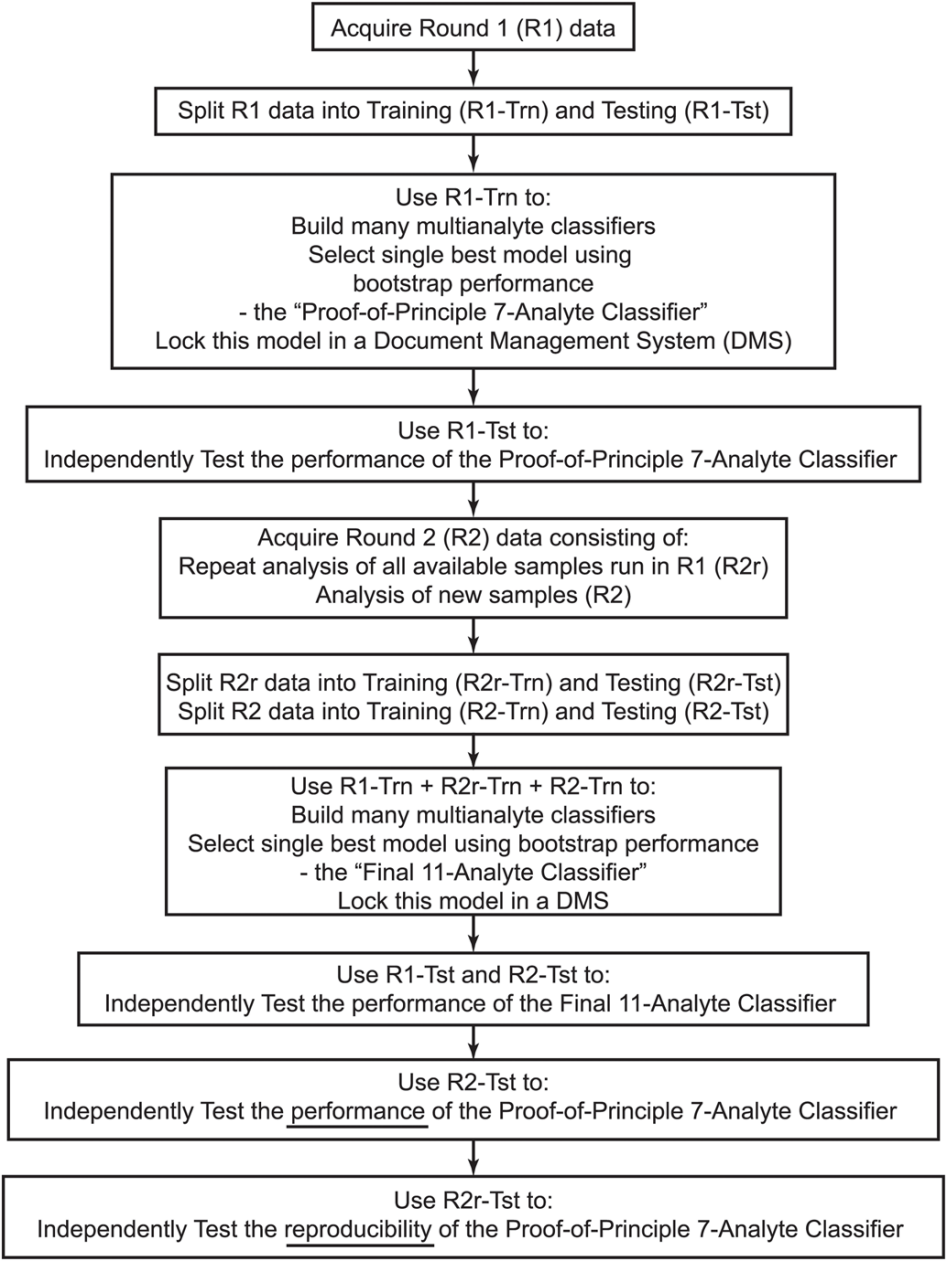
Workflow for model building and testing.

Initially, modeling was performed with data generated in round one (Figure 1) using a modification of Breiman's Random Forest code [28]. The method was improved by enabling batch automation, adding an external layer of bootstrapping, providing greater control over run parameters, and customizing output. The resulting trees were saved and a proprietary routine was used to score samples and output sample information, probability scores, and classification result. Forty stage I ovarian cancer and 40 control samples were used for model building. The controls were selected to ensure that the modeling set represented the same proportions of normal, benign and other cancer conditions as the whole control set, however, within each of those categories, samples were selected randomly. Modeling was optimized by varying both the tree counts (50, 100, 500 and 1000) in a forest, and the number of biomarkers (5, 10, 15, 20, 25, 30, 35, 40, 45, 50) explored at each branching point, resulting in 40 models. From these models, the 20 most informative analytes were identified using the variable importance value. In the second step, a series of models were built that were restricted to the most important analyte (1-analyte model), the two most important analytes (2-analyte model) and so on to a 20-analyte model, a total of 20 models. The OOB and external bootstrap errors, and their standard deviations, were tabulated for each of these models. From these results it was determined that a minimum of seven analytes were required to achieve the most accurate classification. A final, single, model was then built on these seven analytes and deposited into the DMS as a “locked” model.

### Modeling – Final Classifier

The final modeling incorporated all stage I cancer data from rounds one and two, including the duplicates – a total of 91 stage I data sets, representing 61 unique samples and an identical number of controls, matched as before, and balanced in the same round one to round two ratio (Figure 1). Only these data sets (i.e. the training set) were used in model building and selection. The pattern analysis was performed using a unique, patent-pending algorithm, Knowledge Discovery Engine-VS (KDE-VS™). KDE-VS utilizes a group of voting structures similar to decision trees with a unique method of building and defining the cut-off values within each voting structure, using not only the measured value of an analyte but also the laboratory-based error estimate associated with that measurement, derived from the historical QC measurements for each analyte. The user can vary the fractional value of the error estimate incorporated into a classifier during modeling. The result is a robust classifier that can withstand significant perturbation of experimentally determined point values of analyte concentrations. During model building, each terminal node on the voting structure is assigned to a given state – either ovarian cancer or non-ovarian cancer. To score an unknown, our software extracts the values for the analytes of interest to determine which node the sample falls in.

Two different modeling runs, with fractional value of error tolerances of 1.0 and 3.0, were performed using data for the 104 antigen assays. The 20 most robust analytes were determined for each run and these were then assembled into an exhaustive set of 7-marker models. However, all models were required to contain an invariant core of the three most robust and informative analytes, namely CA-125, C-reactive protein and EGF-R, which reduced the search space to 2380 combinations. For both levels of error tolerance we identified the ten most sensitive and ten most specific models – giving a total of 40 models. The frequency of use of both individual analytes and various analyte combinations across all 40 models, led to the identification of 11 analytes that together appeared robust and informative. Finally, a single model was built on these 11 analytes and locked in the DMS. Only after locking the model were the remaining data, not used in training, scored to test the model (Figure 1).

### Data Analysis

Confidence intervals were calculated using the Newcombe method [29].

## Results

### Preliminary Evaluation of the Proof-of-Principle Classifier

The first set of data, generated on 147 ovarian cancer and 149 non-ovarian cancer control samples was used to explore the potential of using a high throughput multiplex immunoassay platform as a discovery tool. We hypothesized that a classification pattern for stage I ovarian cancer would persist through all later stage diseases, so only stage I cancer samples were used for model development. This approach also balanced the average age of case and control patients, removing age-related bias during modeling (Table 1). Through several rounds of enrichment for the most informative biomarkers, driven by the assessment of bootstrap errors for the model development sample set, a 7-analyte model evolved, consisting of CA-125, EGF-R, C-reactive protein, apolipoproteins CIII and A1, IL-18 and tenascin C. This stage I specific profile was locked into the DMS. Only after the model was locked into the DMS was the data for the testing samples (those not used in modeling) accessed and scored by the model to give the results described below (Figure 1).

Since all stage I data generated in the first round of assays had been used in modeling, there were no independent data to test stage I sensitivity. However, the 100-fold bootstrap estimate of stage I sensitivity was 87% (Table 2). The bootstrap estimate for specificity, based on the controls used in model development was 82.3%. The classifier was then evaluated using round one testing samples, a set of independent samples not used in any aspect of model development. The classifier had 95.3% sensitivity and 70.6% specificity. Performance for benign samples was lower (67.1%) than other controls. There was no single subtype of cancer that scored significantly different from the others and when broken down by stage, the sensitivity varied little (94.0–100%), supporting the hypothesis that a stage I pattern could persist through all stages of disease. Following the second round of assays, all round two data were scored on this locked model. The samples common to round one showed a reproducible performance with 97.1% sensitivity (95% CI, 91.0–99.2%) and 74.5% specificity (95% CI, 64.7–82.4%). The additional 69 samples, not previously analyzed, provided a second testing set and yielded 85.7% sensitivity for stage I, 100% sensitivity for stage II and 67.5% specificity.

**Table 2:** Preliminary Performance Evaluation of the 7-Analyte Proof-of-Principle Classifier.

State	Stage or subtype	Testing – Round One	Testing – Round Two	
		Correct/Total (%)	95% CI	Correct/Total (%)	95% CI
**Ovarian Cancer**	**Stage I**	-/40 (87.0*)	N/A	18/21 (85.7)	62.6–96.2
	**Stage II**	22/23 (95.7)	76.0–99.8	8/8 (100)	59.8–100
	**Stage III**	63/67 (94.0)	84.7–98.1	-	-
	**Stage IV**	12/12 (100)	69.9–100	-	-
	**Stage X**	5/5 (100)	46.3–100	-	-
	**Combined**	102/107# (95.3)	88.9–98.3 #	26/29 (89.7)	-
**Non-Ovarian Cancer**	**Benign**	49/73 (67.1)	55.0–77.4	24/36 (66.7)	48.9–80.9
	**Normal**	19/24 (79.2)	57.3–92.1	3/3 (100)	31.0–100
	**Other Cancers**	9/12 (75.0)	42.8–93.3	0/1 (0)	0–94.5
	**Combined**	77/109 (70.6)	61.0–78.8	27/40 (67.5)	50.8–80.9

* The 40 round one stage I samples were used in model development, therefore results for stage I samples are estimates based on 100-fold bootstrap validation.

# stage I values are not included in these calculations, all other samples listed in the table were not used in developing the proof-of-principle model. Correct/Total, the number of samples correctly classified / the total number of samples for each sample type; 95% CI, 95% confidence interval for the result; N/A, not applicable. Stage X, staging not available.

### Preliminary Evaluation of the Final Classifier

The proof-of-principle classifier confirmed our hypothesis that using only stage I data for both model development and assessment we could identify an informative pattern that may exist and persists through later stages of cancer. Therefore, we sought to develop the stage I model further using all stage I samples available. The same modeling strategy was repeated with two important modifications. First, a different, proprietary algorithm was implemented, and second, all stage I samples analyzed across both rounds one and two were used to increase the size of the model development data set (Figure 1). The modeling strategy went through several iterative steps to enrich for the most informative biomarkers, based on an assessment only of stage I training data before culminating in a near-exhaustive search of biomarker combinations that generated 2380 models. Forty models were selected based on their bootstrap sensitivity and specificity on the stage I sample set. By comparing the biomarker combinations in these top 40 models (Table 3), and considering the balance they showed in bootstrap accuracy, sensitivity, specificity, and standard deviations, a final set of 11 informative biomarkers were identified. Certain analyte combinations were common in many models, and there were clearly “substitution patterns” where a different analyte or combination of analytes could yield equivalent models. The 11 biomarkers – CA-125, C-reactive protein, EGF-R, CA 19-9, apolipoproteins A1 and CIII, myoglobin, MIP-1α, IL-6, IL-18 and tenascin C – were assembled into a final model using the KDE-VS algorithm and locked into the DMS as the final model (Figure 1).

**Table 3:** Biomarkers in the Ten Most Specific and Sensitive 7-Marker Models Using a Noise Parameter of 1.0*.

Biomarkers	Model Number																			
	1	2	3	4	5	6	7	8	9	10	11	12	13	14	15	16	17	18	19	20
**CA-125** #	x	x	x	x	x	x	x	x	x	x	x	x	x	x	x	x	x	x	x	x
**CRP** #	x	x	x	x	x	x	x	x	x	x	x	x	x	x	x	x	x	x	x	x
**EGF-R** #	x	x	x	x	x	x	x	x	x	x	x	x	x	x	x	x	x	x	x	x
**CA 19-9**	x	x	x	x	x	x	x	x	x		x	x	x	x	x	x	x	x	x	x
**SAP**			x			x			x											
**Apo A1**							x		x											
**IL-6**			x	x				x		x								x		x
**Myoglobin**					x					x	x	x		x	x	x	x	x	x	x
**MIP-1α**	x	x	x	x			x	x	x	x	x	x	x			x				
**vWF**		x		x			x													
**Leptin**																x	x			
**Apo CIII**					x	x													x	
**GH**											x		x	x	x		x	x		
**IL-18**	x				x	x		x				x		x					x	x
**MPO**										x			x							
**VCAM-1**	x	x													x					

* A comparable list was generated for a noise parameter of 3.0; x, biomarker used in a given model.

# All models were required to contain an invariant core of the three most robust and informative analytes, namely CA-125, C-reactive protein and EGF-R. CA, cancer antigen; CRP, C-reactive protein; EGF-R, soluble epidermal growth factor receptor; SAP, serum amyloid P; Apo, apolipoprotein; MIP-1α, macrophage inhibitory protein 1α; EN-RAGE, Protein S100-A12; CK-MB, creatine kinase-MB; vWF, von Willebrand Factor; GH, growth hormone; MPO, myeloperoxidase; VCAM-1, vascular cell adhesion molecule 1.

As a preliminary test of the classifier's performance, all data not used in model development were scored, yielding 91.3% sensitivity and 88.5% specificity (Table 4, Figure 1). Notably, stage II sensitivity was 83.9% and performance on the benign samples improved to 90.4%. Additional stage I samples were not available, at that time, for testing of this performance. However, the bootstrap estimate of sensitivity for the training set was 83.4% for stage I disease and 84.2% (±12.5%) specificity (Table 4). As a separate exercise, all duplicate data from round two not used in model development were scored. As anticipated from the previous results, the performance was similar with 96.1% sensitivity (95% CI, 89.7–98.7%) and 88.1% specificity (95% CI, 80.8–93.0%) with benign samples scoring 87.0% (95% CI, 76.2–93.5%). To provide a frame of reference, we compared the model performance to that of a clinical decision based on CA-125 expression levels. Since the cut-off value of 35 IU/ml is already established, the complete data set was used to assess the predictive value of CA-125. With this cut-off value, CA-125 gave 94.9% sensitivity and 58.6% specificity (Table 5). For stage I samples alone, the sensitivity dropped to 88.5%.

**Table 4:** Preliminary Evaluation of the Final 11-analyte Classifier.

Ovarian Cancer	Number of samples#	Correct classification	% Sensitivity	95% CI
**Stage I**	61	-	83.4% (±12.4%)*	
**Stage II**	31	26	83.9	65.5–93.9
**Stage III**	67	62	92.5	82.7–97.2
**Stage IV**	12	12	100.0	69.8–100
**Unstaged**	5	5	100.0	46.3–100
**Combined** ¶	115	105	91.3	84.2–95.5

Non-Ovarian Cancer	Number of samples#	Correct classification	% Specificity	95% CI
**Benign**	73	66	90.4	80.7–95.7
**Prospective Benign** ‡	20	17	85.0	61.1–96.0
**Normal**	24	21	87.5	66.5–96.7
**Other Cancer**	13	11	84.6	53.7–97.3
**Combined**	130	115	88.5	81.4–93.2

# The final model was used to score all round one data, excluding those used in model development, and all additional samples unique to round two, which were not used in model development.

* the performance for the stage I samples is an estimate based on the 100-fold bootstrap results.

‡ these benign samples are from Correlogic's prospective collection, collected under an IDE to support the development of a clinical test for ovarian cancer. The study population is women scheduled for surgery presenting with symptoms of ovarian cancer. As such, disease status is pathology confirmed following surgery.

¶ stage I values are not included in these calculations. 95% CI, 95% confidence interval for the result.

**Table 5:** Comparison of the Classification Performance of CA-125, the Proof-of-Principle and the Final Classifier.

Method	Sensitivity	95% CI	Specificity	95% CI
**All Samples** * **CA-125>35 IU/ml**	**94.9%**	90.5–97.6	**58.6%**	51.9–65.6
**Stage I only** # **CA-125>35 IU/ml**	**88.5%**	77.8–95.3	**58.6%**	51.9–65.6
**Proof-of-principle 7-analyte classifier** ‡	**94.1%**	88.4–97.2	**69.8%**	61.7–76.9
**Final 11-analyte classifier** ¶	**91.3%**	84.2–95.5	**88.5%**	81.4–93.2

* Since the cut-off value of 35 IU/ml is already established, the complete data set, excluding duplicates was used to assess the predictive value of CA-125.

# sensitivity for all stage I samples only, excluding duplicates.

‡ sensitivity and specificity determined using the 136 ovarian cancer and 149 non-ovarian cancer samples, excluding duplicates, not used in development of the 7-anlayte classifier.

¶ sensitivity and specificity determined using the 115 ovarian cancer and 130 non-ovarian cancer samples, excluding duplicates, not used in development of the 11-anlayte classifier. 95% CI, 95% confidence interval for result.

We implemented two methods to estimate the importance of the different analytes to the overall classifier. First, we assessed model performance when all but one analyte was held constant in the data files, with the value of the chosen analyte randomized. This was repeated sequentially for each analyte. The relative value of each analyte was then ranked by determining which analyte caused classification performance to decline the most when randomized. We observed that biomarker importance tended to group together. Specifically, CA-125 was the most important biomarker, followed a group consisting of C-reactive protein, CA 19-9 and EGF-R, followed by MIP-1α, followed by myoglobin, apolipoprotein CIII, apolipoprotein A1, IL-18 and IL-6 and finally tenascin C. As a second method of estimating analyte importance, we analyzed the branching points of the voting structures. Across all branching points of the voting structures, CA-125 was involved the most frequently (15.8%) followed by CA 19-9 (12.1%), myoglobin (11.1%), C-reactive protein (10.8%) and EGF-R (9.9%). CA-125 was utilized in 80% of the top-level branching points, representing the first major sample partitioning, followed by C-reactive protein (11.2%), EGF-R (5.0%) and CA19-9 (1.8%). At the second tier, CA19-9 was used most frequently (20.3%) followed by EGF-R (18.8%), CA-125 (11.4%), myoglobin (9.8%), tenascin C (8.0%), IL-18 (7.2%), and apolipoprotein A1 (6.9%). The acute phase markers MIP-1α and IL-6 were seen only 6.2% and 1.3% respectively at this level.

## Discussion

In this study we identified a classification pattern for ovarian cancer in the serum proteome of patients with stage I disease, which remains evident through later stage disease. Sera from patients with pathologist-confirmed conditions – either with or without epithelial ovarian cancer – were profiled using a bead-based multi-analyte profiling approach. The analytes covered a broad range of biological structures and functions, including cancer antigens, hormones, clotting factors, tissue modeling factors, lipoprotein constituents, proteases and protease inhibitors, markers of cardiovascular risk, growth factors, cytokine/chemokines, soluble forms of cell-signaling receptors, and inflammatory and acute phase reactants as well as markers for autoimmunity and infection (Table S1). Two independent analyses of samples were performed 86 days apart. There were several reagent lot and batch changes during this period, providing a real world challenge to the robustness of the underlying assays and the model.

**Figure 2:**
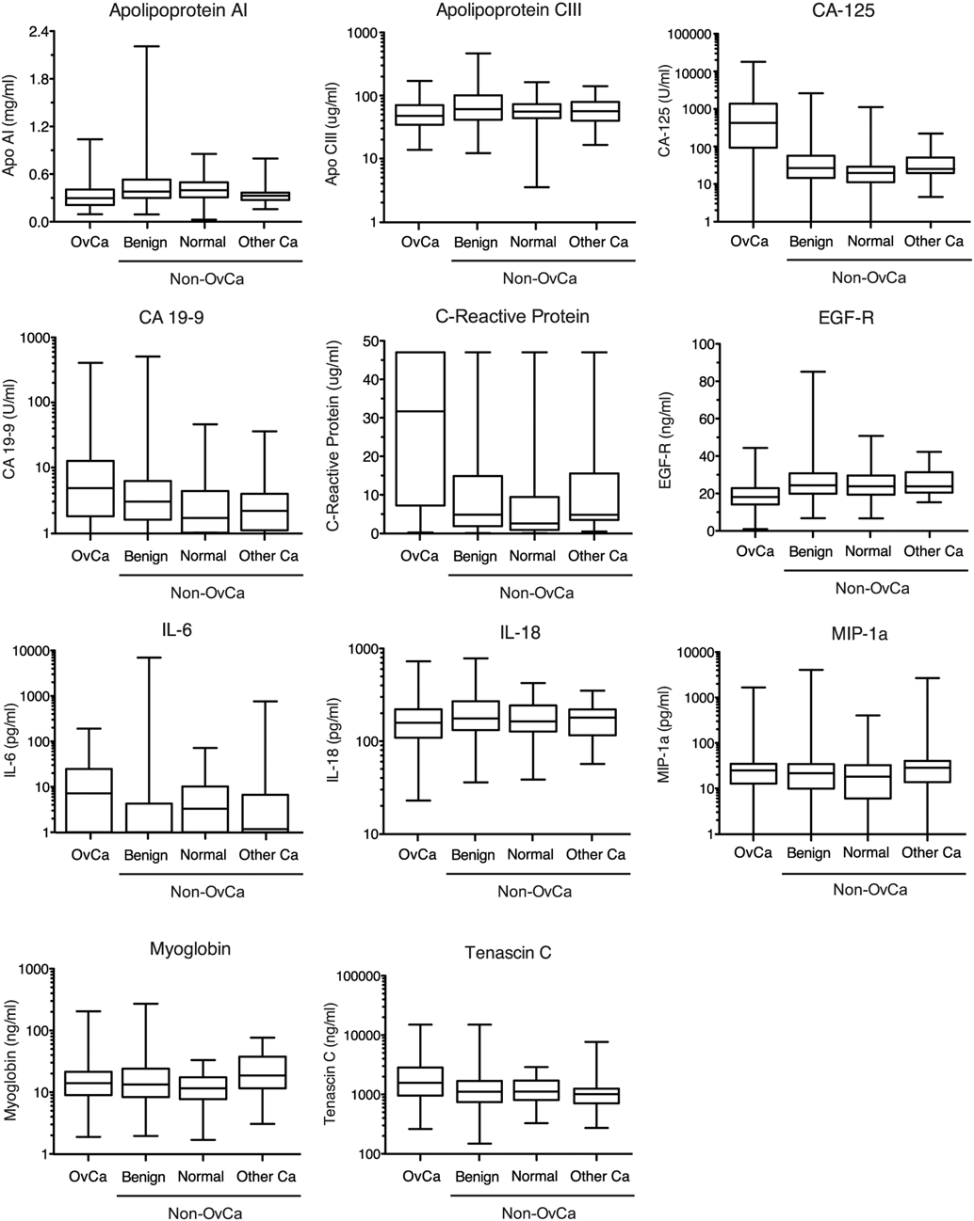
Serum level distributions for the analytes used in the final 11-analyte classifier. For each analyte, the box-whisker plots show: the lowest observation, lower quartile, median value, upper quartile, and highest observation. All analyses, including duplicates are shown. CA-125 – one ovarian cancer, 11 benign and five normal samples below lowest calibration value; CA 19-9 – 14 ovarian cancer, 18 benign, nine normal and four other cancer samples below lowest calibration value; C-reactive protein – 93 ovarian cancer, 21 benign, two normal and two other cancer samples above highest calibration value; IL-6 – 82 ovarian cancer, 161 benign, 28 normal and 14 other cancer samples below lowest calibration level; MIP-1α – 50 ovarian cancer, 53 benign, 10 normal and four other cancer samples below lowest calibration level; tenascin C – two ovarian cancer and one benign sample above highest calibration level. OvCa, ovarian cancer; Ca, cancer; Apo, apolipoprotein; CA-125, cancer antigen 125; CA 19-9, cancer antigen 19-9; EGF-R, epidermal growth factor receptor (soluble form); IL, interleukin; MIP-1a, macrophage inflammatory protein 1 alpha.

Four major components were critical to the success of this study. First, it was essential to identify a highly consistent, well-documented and clinically representative sample set of confirmed cases and controls. For ovarian cancer, confirmation can only come from pathologic examination of surgically excised tissue. We selected serum samples from well-characterized collections from women already scheduled for surgery. The substantial majority of controls in this population had pathology-confirmed benign conditions, which based on univariate analysis, should pose a greater challenge for classification than sera from non-symptomatic women (Figure 2; [24]). Second, we utilized a panel of fully qualified, high throughput, immunoassays that measure a wide diversity of molecules including autoimmune and infectious disease markers, and a wide range of well characterized serum proteins, including those previously implicated in ovarian cancer. Third, we used a novel multivariate modeling approach to identify a robust pattern of molecules informative for ovarian cancer. The proprietary algorithm (KDE-VS) improved classification performance compared to Random Forest and other classification algorithms by building robust decision boundaries into its voting structures, which incorporates real-world experimental variability into the data being modeled. Finally there was a clear separation between samples used to develop and identify a single informative model, and the samples used to evaluate that models performance.

Our study focused on the analysis of early stage disease with >50% of the cancer sample set representing stages I and II disease (Table 1). Consistent with the literature, the average patient age at diagnosis correlated with the stage of disease at diagnosis (Table 1; [22]). The subtype distribution was representative of the US population, with a larger proportion of serous (42%) and endometrioid (26%) carcinoma (Table 1). The control samples were predominantly from individuals with common benign ovarian conditions (75%), as well as other gynecological and non-gynecological cancers (8%), and a small number of non-diseased samples (17%), consistent with the need for a clinical test for symptomatic women (Table 1).

Our rationale to focus on early stage disease was two-fold. Firstly, early stage ovarian cancer is considered curable, but in many cases symptoms are subtle and hard to detect. If there is an informative pattern in stage I disease, it would be useful to identify for later validation. Secondly, we sought to minimize the impact of CA-125 on the development of any potential classification pattern. It is widely accepted that CA-125 is more elevated in late stage disease than early stage disease, and is the most strongly correlated single biomarker for ovarian cancer at any stage [24], although it lacks specificity. By examining stage I disease, where CA-125 may be less dominant, we hypothesized that other informative proteomic combinations might be discovered and that these would persist through later disease stages. Our results support these assumptions for the sample set studied. Indeed, in later work (unpublished) in which we built classification models using later disease stages for the model development set, we did identify patterns strongly predictive of ovarian cancer. However, the patterns were dominated by CA-125 and had poor performance when evaluated on early stage disease samples.

All stage I samples were devoted to model development to maximize the training sample set size. Therefore, a weakness of this study is the lack of samples to test independently the performance on stage I disease. Bootstrap estimates have proven to be good indicators in our model building to date. In the proof-of-principle classifier, bootstrap estimates predicted the 7-biomarker model would have an accuracy of 87% on stage I samples. This was supported when independent round two, stage I samples were scored by the model with an 85.7% accuracy. For the final modeling run that generated the 11-analyte model, the bootstrap estimate predicted a stage I sensitivity of 83.4%. While our strategy involved several steps and training data were used repetitively to refine the set of the most informative assays it is critical to appreciate that only the model development set, composed of the stage I data and an equal number of non-ovarian cancer data, were used repetitively. The other data were never used until a final model was locked into the DMS for final testing. Therefore the performance characteristics we observed for all non-stage I cancer and non-ovarian cancer samples not used in model building are independent results.

To assess the impact of sample bias on our results, we examined three potential sources of concern. Age is a risk factor for ovarian cancer, and could therefore introduce bias caused by age or menopausal differences between the cases and controls. We addressed this in several ways. Firstly, we used a strategy in which the average age of cases and controls were very similar in the model development set. Secondly, in a separate modeling analysis we reorganized the model development set into two groups divided by age (≤50 years versus ≥61 years), irrespective of disease status. Interestingly, infectious disease markers were the predominant predictors of age, perhaps reflecting different vaccination or exposure histories. A similar type of analysis was undertaken to address the different length of storage of individual sera in the −80°C freezer. This did not give statistically significant classification. Finally, we attempted to build classifiers for samples that were completely randomized regardless of disease status and again, no statistical significant multivariate classifiers could be generated.

Only after this modeling had been completed, the performance characteristics of individual markers were determined on the sample set used in round one of this study [24]. Interestingly, the combination of markers in the final model is not the combination that would be selected from the best individual analytes. Indeed most of the selected markers provide little classification value for cancer status when considered alone as individual markers (Figure 2; [24]) with only CA-125 and C-reactive protein having appreciable classification potential. The markers in the 11-analyte classifier reflect a variety of biological functions. However, two cancer antigens, CA-125 and CA 19-9, along with EGF-R, a truncated signaling receptor associated with cell growth and differentiation, and the inflammatory marker C-reactive protein are involved in the majority of initial decisions in the voting structure and primarily drive the performance of the classifier. The remaining markers are cytokines (IL-18, IL-6 and MIP-1α), metabolic markers (apolipoproteins A1 and CIII), myoglobin (an oxygen carrier) and tenascin C (an extracellular matrix protein). While it is difficult to predict the particular biological roles of the markers that contribute to the ovarian cancer pathology, they are all implicated in multiple pathways associated with tumor growth and metastasis. In this context it is interesting to note that the combination of both CA-125 and CA 19-9 provide complimentary information for non-mucinous (CA-125 elevated) and mucinous (CA 19-9 elevated) cancers [30]. One other immediate observation is the implication of three proteins (C-reactive protein, IL-6, MIP-1α) commonly associated with an acute phase response. While it has been proposed that up to 23% of ovarian cancers have a chronic inflammatory component [31], it is notable that the relative importance of IL-6 and MIP-1α to other analytes in the 11-analyte classifier is low, reflected both by the calculated importance value, and the absence of IL-6 and MIP-1α in the 7-analyte classifier. Since both IL-6 and MIP-1α are multifunctional proteins, it is difficult to know if their biological roles are purely inflammatory, or more complex.

The selection of myoglobin in the final 11-analyte classifier was not intuitive. It is primarily considered a marker of muscle damage and the underlying biological role that myoglobin plays in ovarian cancer is unclear. Indeed, myoglobin levels do not appear to differ significantly between the ovarian cancer and control samples [24] yet our analysis showed it has a relatively strong contribution to the classifier. Analysis of the voting structures indicates that myoglobin, as well as tenascin C, are often used as a terminal decision point forming the final decisions on whether a sample is classified as a case or control. This may reflect a role in normalizing the relative expression levels for the other analytes.

In our method of model development it is not possible to fix a pre-desired sensitivity or specificity for a classifier. Therefore, we have not presented an ROC curve. In an ROC curve built upon a single variable (such as CA-125), the cut-off values on the curve reflect the analyte concentrations measured. In this instance, changing cut-offs for a given sensitivity and specificity is very practical. In a multivariate index, there is a large dimensional reduction interpreting multiple analyte concentrations into a single value which now represents an index value. In the case of a regression equation of the form ax+by, the same index value can be achieved by many different combinations of x and y. As the number of parameters in the expression increase, so do the combinations. A similar effect occurs in our voting structures where many different combinations of voting structures can lead to a similar overall vote. Moreover, the way the voting structures are built depends upon a set of decision rules, which guide their evolution. These rules are intimately tied into how a branch point in the voting structure is defined, and therefore changing the ROC cut-off after a model is built is not valid. Any cut-off that does not reflect the rules used to create the models during model development will not be robust. However, the shape and AUC of the ROC are useful envisioning and comparing the overall accuracy of different multivariate indexes. An ROC curve generated on the final 11-analyte model, using only samples not used in model development, to avoid over-fitting, yielded an area underneath the curve (AUC) value of 0.953, significantly better than CA-125 alone [24].

Women who are suspected of having ovarian cancer require a thorough clinical assessment to determine their risk for ovarian cancer. Many women present with benign pelvic masses that are treated effectively by surgical recision, under the care of an obstetrician–gynecologist or general surgeon. When a pelvic mass proves to be a malignant neoplasm, formal staging and thorough surgical resection is required to achieve an optimal likelihood of cure [32]. Therefore it is clinically useful to have a test that will lead women with a high likelihood of having ovarian cancer into the care of a gynecologic oncologist [33] while taking care not to over-refer benign conditions. Guidelines to help assess and triage patients have been addressed by the American College of Obstetricians and Gynecologists (ACOG) and the Society of Gynecologic Oncologists (SGO), however strict adherence to these guidelines is often incomplete. A blood-based test that could improve this triage would be of benefit [32]. The 11-analyte classification pattern described in this paper has characteristics consistent with this use, but requires a statistically significant clinical validation on a validated, custom multiplex, to verify its performance.

There are a number of limitations to our study. Firstly, the performance of the classifier can only be considered preliminary and is likely over-optimistic because of the nature of the testing set. The testing samples were sourced from the same collection, and analyzed at the same time as those used in model building (training). Therefore, the performance on a truly independent set (i.e. from different sources and analyzed at a different time point) is likely to be lower. Validation on a totally independent set must be conducted and this forms the basis of our ongoing studies. Secondly, our study is specifically focused on epithelial ovarian cancers. We intentionally excluded non-epithelial ovarian cancer subjects because they are rare, and it would be difficult to identify sufficient numbers for a statistically sound study. Thirdly, we limited the number of low malignant potential (LMP) tumors because there are differences of opinion on how to classify LMP tumors. In order to establish a clear hypothesis we focused on the classification of pathology proven epithelial ovarian cancers from pathology proven ovarian benign conditions. As such, the performance of the test in our preliminary evaluation can not be generalized to a clinical population, which would require an independent validation study.

## Supporting Information

Table S1 Assays Performed on Samples. The antigen, autoimmune and infectious disease panels consisted of the following assays.
(0.04 MB DOC)

Table S2 Sources of Specimens by Collection Site.
(0.08 MB DOC)
