# Supplementary material for: Development and Preliminary Evaluation of a Multivariate Index Assay for Ovarian Cancer
Source: PLoS One. 2009 Feb 25;4(2):e4599. doi: 10.1371/journal.pone.0004599 (PMC2643010; doi:10.1371/journal.pone.0004599)
Supplement: Table S1 — Assays Performed on Samples. The antigen, autoimmune and infectious disease panels consisted of the following assays. (0.04 MB DOC) [file pone.0004599.s001.doc]

**Table S1.** Assays Performed on Samples. The antigen, autoimmune and infectious disease panels consisted of the following assays.

| **Panel** | **Markers** |
| --- | --- |
| **Antigens** | Adiponectin, α-1 anti-trypsin, α-2 macroglobulin, α-fetoprotein, amphiregulin, apolipoprotein A1, apolipoprotein CIII, apolipoprotein H, β-2 microglobulin, betacellulin, brain-derived neurotrophic factor, calcitonin, cancer antigen 125, cancer antigen 19-9, carcinoembryonic antigen, CD40, CD40 ligand, complement 3, connective tissue growth factor, c-reactive protein, creatine kinase-MB, endothelin-1, eotaxin, epidermal growth factor, epidermal growth factor receptor, epiregulin, epithelial-derived neutrophil-activating protein 78, erythropoietin, factor VII, fatty acid binding protein, ferritin, fibrinogen, fibroblast growth factor basic, glutathione s-transferase, granulocyte colony-stimulating factor, granulocyte-macrophage colony-stimulating factor, growth hormone, haptoglobin, heparin-binding EGF-like growth factor, interferon-γ, immunoglobulin A, immunoglobulin E, immunoglobulin M, insulin, insulin-like growth factor 1, intercellular adhesion molecule 1, interleukin-1α, interleukin-1β, interleukin-10, interleukin-12p40, interleukin-12p70, interleukin-13, interleukin-15, interleukin-16, interleukin-17, interleukin-17e, interleukin-18, interleukin-1ra, interleukin-2, interleukin-23, interleukin-3, interleukin-4, interleukin-5, interleukin-6, interleukin-7, interleukin-8, leptin, lipoprotein (a), lymphotactin, macrophage inflammatory protein-1α, macrophage inflammatory protein-1β, macrophage-derived chemokine, matrix metalloproteinase-2, matrix metalloproteinase-3, matrix metalloproteinase-9, monocyte chemotactic protein-1 , myeloperoxidase, myoglobin, oncostatin M, plasminogen activator inhibitor-1, platelet-derived growth factor, pregnancy-associated plasma protein A, prostate specific antigen (free), prostatic acid phosphatase, protein S100-A12 (EN-RAGE), Regulated upon Activation, Normal T-cell Expressed, and Secreted (RANTES), serum amyloid P, serum glutamic oxaloacetic transaminase, sex hormone binding globulin, stem cell factor, tenascin C, thrombopoietin, thrombospondin-1, thyroid stimulating hormone, thyroxine binding globulin, tissue factor, tissue inhibitor of metalloproteinases-1, transforming growth factor-α, tumor necrosis factor RII, tumor necrosis factor-α, tumor necrosis factor-β, vascular cell adhesion molecule 1, vascular endothelial growth factor, von willebrand factor |
| **Autoimmune** | Anti-nuclear antibody, ASCA (saccharomyces cerevisiae) antibody, β-2 glycoprotein antibody, centromere protein B antibody, collagen type 1 antibody, collagen type 2 antibody, collagen type 4 antibody, collagen type 6 antibody, complement factor C1q antibody, cytochrome p450 antibody, double stranded DNA antibody, histone antibody, histone H1 antibody, histone H2A antibody, histone H2B antibody, histone H3 antibody, histone H4 antibody, heat shock cognate 70 antibody, heat shock protein 32 (ho) antibody, heat shock protein 65 antibody, heat shock protein 71 antibody, heat shock protein 90 α antibody, heat shock protein 90 β antibody, insulin antibody, JO-1 (histidyl tRNA synthetase) antibody, mitochondrial antibody, myeloperoxidase (PANCA) antibody, pancreatic islet cells (GAD) antibody, PCNA (proliferating cell nuclear antigen) antibody, polymyositis-1 antibody, proteinase 3 (CANCA) antibody, ribosomal P antibody, ribonucleoprotein (a) antibody, ribonucleoprotein (c) antibody, ribonucleoprotein antibody, scleroderma-70 antibody, Smith antibody, SSA (ro) antibody, SSB (la) antibody, T3 (triiodothyronine) antibody, T4 (thyroxine) antibody, thyroglobulin antibody, thyroid microsomal antibody, TTG (tissue transglutaminase celiac disease) antibody |
| **Infectious disease** | Adenovirus, Bordetella pertussis, Campylobacter jejuni, Chlamydia pneumoniae, Chlamydia trachomatis, Cholera Toxin, Cholera Toxin - subunit B, Cytomegalovirus, Diphtheria Toxoid, Epstein Barr virus - early antigen, Epstein Barr virus - nuclear antigen, Epstein Barr virus VCA, Helicobacter pylori , hepatitis A, hepatitis B - core, hepatitis B - envelope, hepatitis B - surface (Ad), hepatitis B - surface (Ay), hepatitis C - core, hepatitis C - NS3, hepatitis C - NS4, hepatitis C - NS5, hepatitis D, hepatitis E - orf2 3kd, hepatitis E - orf2 6kd, hepatitis E - orf3 3KD, herpes simplex virus-1 gD, herpes simplex virus-1/2, herpes simplex virus-2 gG, human immunodeficiency virus gp120, human immunodeficiency virus gp41, human immunodeficiency virus p24, human papilloma virus, human T-cell lymphotropic virus 1/2, influenza A, influenza A - H3N2, influenza B, Leishmania donovani , Borrelia burgdorferi (Lyme), Mycoplasma pneumoniae, Mycobacterium tuberculosis, mumps virus, parainfluenza 1, parainfluenza 2, parainfluenza 3, polio virus, respiratory syncytial virus, Rubella, Rubeola, Streptolysin O (SLO), Trypanosona cruzi, Treponema pallidum 15kd, Treponema pallidum p47, Tetanus toxoid, Toxoplasma, Varicella zoster (Chickenpox). |
