# Supplementary material for: Development and Preliminary Evaluation of a Multivariate Index Assay for Ovarian Cancer
Source: PLoS One. 2009 Feb 25;4(2):e4599. doi: 10.1371/journal.pone.0004599 (PMC2643010; doi:10.1371/journal.pone.0004599)
Supplement: Table S2 — Sources of Specimens by Collection Site. (0.08 MB DOC) [file pone.0004599.s002.doc]

**Table S2.** Sources of Specimens by Collection Site.

| **Site Number*** | **Non-OvCa** | **OvCa** | **Site Number*** | **Non-OvCa** | **OvCa** |
| --- | --- | --- | --- | --- | --- |
| **1** | 8 | 6 | **28** | 1 | 3 |
| **2** | 1 | 2 | **29** | 0 | 4 |
| **3** | 0 | 3 | **30** | 0 | 1 |
| **4** | 4 | 1 | **31** | 0 | 2 |
| **5** | 2 | 1 | **32** | 1 | 0 |
| **6** | 7 | 2 | **33** | 0 | 1 |
| **7** | 0 | 3 | **34** | 1 | 1 |
| **8** | 12 | 7 | **35** | 2 | 0 |
| **9** | 0 | 3 | **36** | 1 | 0 |
| **10** | 1 | 2 | **37** | 3 | 1 |
| **11** | 0 | 1 | **38** | 4 | 1 |
| **12** | 1 | 0 | **39** | 1 | 0 |
| **13** | 0 | 2 | **40** | 4 | 1 |
| **14** | 1 | 0 | **41** | 1 | 0 |
| **15** | 4 | 1 | **42** | 1 | 1 |
| **16** | 1 | 0 | **43** | 13 | 4 |
| **17** | 1 | 0 | **44** | 11 | 4 |
| **18** | 0 | 1 | **45** | 0 | 1 |
| **19** | 1 | 2 | **46** | 3 | 9 |
| **20** | 3 | 9 | **47** | 0 | 4 |
| **21** | 39 | 44 | **48** | 0 | 2 |
| **22** | 3 | 2 | **49** | 5 | 7 |
| **23** | 7 | 3 | **50** | 5 | 0 |
| **24** | 27 | 16 | **51** | 1 | 0 |
| **25** | 1 | 0 | **52** | 2 | 12 |
| **26** | 0 | 1 | **53** | 1 | 4 |
| **27** | 0 | 1 | **54** | 2 | 0 |

* Encoded identifier for collection site.
